# Efficacy of Digital Outreach Strategies for Collecting Smoking Data: Pragmatic Randomized Trial

**DOI:** 10.2196/50465

**Published:** 2024-02-09

**Authors:** Lauren E Kearney, Emily Jansen, Hasmeena Kathuria, Katrina Steiling, Kayla C Jones, Allan Walkey, Nicholas Cordella

**Affiliations:** 1 The Pulmonary Center Boston University Boston, MA United States; 2 Department of Quality and Patient Safety Boston Medical Center Boston, MA United States; 3 The Evan's Center for Implementation & Improvement Sciences Boston University Boston, MA United States

**Keywords:** electronic health records, EHR, informatics, learning health system, lung cancer screening, smoking history

## Abstract

**Background:**

Tobacco smoking is an important risk factor for disease, but inaccurate smoking history data in the electronic medical record (EMR) limits the reach of lung cancer screening (LCS) and tobacco cessation interventions. Patient-generated health data is a novel approach to documenting smoking history; however, the comparative effectiveness of different approaches is unclear.

**Objective:**

We designed a quality improvement intervention to evaluate the effectiveness of portal questionnaires compared to SMS text message–based surveys, to compare message frames, and to evaluate the completeness of patient-generated smoking histories.

**Methods:**

We randomly assigned patients aged between 50 and 80 years with a history of tobacco use who identified English as a preferred language and have never undergone LCS to receive an EMR portal questionnaire or a text survey. The portal questionnaire used a “helpfulness” message, while the text survey tested frame types informed by behavior economics (“gain,” “loss,” and “helpfulness”) and nudge messaging. The primary outcome was the response rate for each modality and framing type. Completeness and consistency with documented structured smoking data were also evaluated.

**Results:**

Participants were more likely to respond to the text survey (191/1000, 19.1%) compared to the portal questionnaire (35/504, 6.9%). Across all text survey rounds, patients were less responsive to the “helpfulness” frame compared with the “gain” frame (odds ratio [OR] 0.29, 95% CI 0.09-0.91; *P*<.05) and “loss” frame (OR 0.32, 95% CI 11.8-99.4; *P*<.05). Compared to the structured data in the EMR, the patient-generated data were significantly more likely to be complete enough to determine LCS eligibility both compared to the portal questionnaire (OR 34.2, 95% CI 3.8-11.1; *P*<.05) and to the text survey (OR 6.8, 95% CI 3.8-11.1; *P*<.05).

**Conclusions:**

We found that an approach using patient-generated data is a feasible way to engage patients and collect complete smoking histories. Patients are likely to respond to a text survey using “gain” or “loss” framing to report detailed smoking histories. Optimizing an SMS text message approach to collect medical information has implications for preventative and follow-up clinical care beyond smoking histories, LCS, and smoking cessation therapy.

## Introduction

Tobacco use is an important risk factor for multiple diseases, including lung cancer, and is one of the leading contributors to preventable death in the United States [1]. The collection of nuanced, complete, and accurate tobacco use histories has significant implications for clinical care. For example, determination of lung cancer screening (LCS) eligibility (eligibility criteria: adults aged between 50 and 80 years with a 20 pack-year smoking history and who are either currently smoking or have quit within the past 15 years) [2] requires full documentation of pack-years (calculated by multiplying the number of packs of cigarettes smoked per day by the number of years the person has smoked) [3]. Clinicians across specialties discuss smoking histories with patients and record them in structured (eg, within a dedicated place in social history) and unstructured data fields (eg, within the clinical note) in the electronic medical record (EMR). Despite this theoretical wealth of longitudinal smoking information, past research illustrates that smoking history data documented in the health record are usually inaccurate, internally inconsistent, incomplete, or outdated [3-8]. Further, while unstructured data may be more accurate, it is limited in its ability to be extracted quickly and easily for clinical care [9,10]. Interventions aimed at improving the documentation and use of patient tobacco histories may have significant implications for interventions that seek to accurately identify patients eligible for LCS, cardiovascular risk reduction, and smoking cessation interventions [4,11,12].

A learning health system (LHS) systematically integrates clinical care, informatics, and research and engages patients to provide opportunities to implement new knowledge rapidly and iteratively [13]. Patient-generated health data (PGHD) is one promising modality to engage patients and build components of a LHS [14]. One challenge, however, is determining the best approach to engaging patients for self-reported data and scaling interventions for use across the health care system. Implementation of initiatives using patient surveys to generate health data are most effective when systematically designed and studied to determine effectiveness and scalability [15,16]. For example, which technology to use and which message framing to use are important considerations to optimize PGHD. Framed messages describe a choice in terms of how participation may provide gain or loss to the individual or helpfulness to the clinician.

We used a PGHD approach to address the issue of poorly documented smoking history, a previously highlighted barrier to uptake of LCS at our institution [7]. Given the clinical relevance of smoking histories to LCS and smoking cessation counseling, the primary objective of this study was to evaluate the impact of patient-generated methods to improve smoking history documentation. To this end, we designed a quality improvement intervention to evaluate three questions about patient-generated smoking history data: (1) “What is the effectiveness of portal questionnaires versus SMS text message–based surveys?” (2) “What is the most effective message framing accompanying the survey link?” and (3), “What is the optimal approach to following up on uncompleted surveys to increase response rates?”

## Methods

### Setting and Cohort

We conducted this trial at a large academic safety net hospital in the northeast United States [17]. The institutional review board determined this project qualified for an exemption determination as quality improvement research. We carried out our pragmatic trial from October 2022 to January 2023 in the general internal medicine practice, the largest adult primary care clinic at our hospital. Our hospital uses EPIC (Verona), referred to as “EMR” throughout this manuscript.

### Participants

A quality analyst generated random patient lists from the EMR for portal questionnaires and text survey cohorts. Our inclusion criteria were a history of tobacco use (current or former), being aged between 50 and 80 years, and having English as a preferred language. We based the patient’s smoking status on their recorded substance use history within the structured social history section of the medical record. In addition to smoking status, this included entry fields for cigarette smoking start date, quit date, cigarette packs per day, cigarette use years, and a pack-year calculated field, which multiplied the packs per day and years. Not all fields were complete for every patient. Because this trial was designed to increase uptake of LCS by gathering an accurate smoking history, we excluded patients who had LCS or an existing LCS order pending since presumably a more accurate smoking status already existed. Additionally, the portal cohort had to have an active portal account, whereas the SMS text message survey cohort needed to have a recorded mobile or home number documented. Finally, we excluded patients from the text survey cohort if they received the portal questionnaire message, so that each cohort was mutually exclusive.

The portal questionnaire cohort consisted of 500 patients, and the text survey cohort consisted of 1000 patients from general internal medicine clinics. The sample size was determined through a judgment sampling approach [18]. Time and resource limitations (our text survey contract was capped at 1000 patients) played a role in the determination of the judgment sample size. The chosen sample size sought to balance meaningful insights and adherence to practical constraints.

### Smoking History Query Interventions

We evaluated 2 modalities: an electronic health record portal questionnaire using EPIC’s MyChart (Verona) and a text survey using Patient Navigation Manager CareTour (Philips Healthcare), a texting platform. The EPIC MyChart questionnaire is referred to as the “portal questionnaire,” and the Philips Healthcare texting platform is referred to as the “text survey” throughout this manuscript. For both modalities, questions pertaining to obtaining an accurate smoking history were designed based on a review of the literature and in consultation with pulmonary and critical care specialists with expertise in tobacco dependence treatment and written in plain language to promote readability and interpretability (Table S1 and Figures S1 and S2 in Multimedia Appendix 1 provide information on the survey questions and user interface). We also used an intentional phased approach for both surveys to assess technical issues, identify remediable issues, and scale more widely. Given that this study used patient-generated smoking history queries, which required participant comprehension and engagement, blinding participants to the study’s purpose was deemed impractical.

#### Message Framing

We reviewed the behavioral economic theory literature to inform our approach and to use message framing that we hoped would best engage patients [15,16,19,20]. We chose to evaluate 3 message framings: “gain,” “loss,” and “helpfulness” (Table 1). Gain- or loss-framed messages have been shown to be effective in smoking cessation, cancer prevention, and vaccination work [21,22]. We also included a helpfulness message, which has been explored within the web-based industry and marketing research but has been underexplored in health care settings [23,24]. We tested these different message frames in the text survey. The EPIC MyChart questionnaire portal system is configured to send users a general email that reads, “You have a portal questionnaire message.” This is a global configuration setting that cannot be modified on a per-project basis. Thus, we did not test different message frames in the portal questionnaire.

#### Survey Modalities

##### Electronic Health Record Portal Questionnaire

The portal questionnaire to assess smoking status consisted of up to 6 questions, which were a combination of multiple-choice or open-ended questions with answers restricted to numeric-only values (Table S1 and Figure S2 in Multimedia Appendix 1). The portal questionnaire design permitted the conditional display of questions tailored to their smoking status. For example, only people who formerly smoked saw the question, “How old were you when you stopped smoking?” Once the message was accessed, a “helpfulness” frame (Table 1) was shown in the portal message’s subject and body (Multimedia Appendix 1 provides full details of surveys).

**Table 1:** Comparison of features tested in portal questionnaire and text survey.

Survey modality	Message faming options	Conditional display of messages	Nudge message
Portal questionnaire (EPIC’s MyChart; Verona)	“Helpfulness” frame for all patients: Help your [hospital name] healthcare team give you the best care possible by answering a few questions about your health.(Msg/data rates may apply. Reply STOP to stop msgs)	Yes	No
Text survey (Patient Navigation Manager CareTour texting platform; Philips Healthcare)	“Gain”: Please answer a few questions for your [hospital name] healthcare team to help you get screenings to keep you healthy. (Msg/data rates may apply. Reply STOP to stop msgs) “Loss”: Please answer a few questions for your [hospital name] healthcare team so you don't miss out on any screenings. (Msg/data rates may apply. Reply STOP to stop msgs) “Helpfulness”: Help your [hospital name] healthcare team give you the best care possible by answering a few questions about your health.(Msg/data rates may apply. Reply STOP to stop msgs)	No	Yes

**Figure 1:**
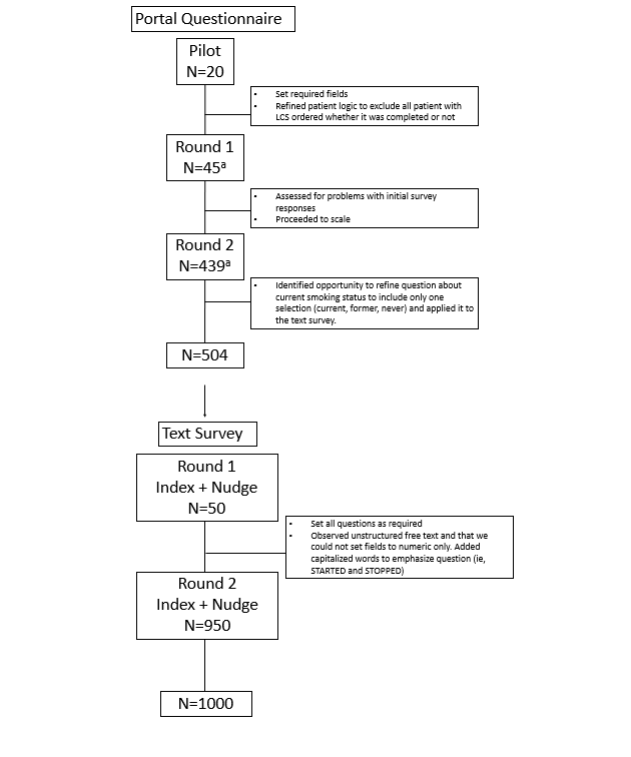
Portal questionnaire and text survey phased rollout and formative modifications. ^a^A total of 16 participants were sent the survey both in the pilot and in the subsequent rounds of the portal questionnaire rollout (5 in round 1 and 11 in round 2). These participants were only included for analysis based on their initial participation in the pilot and were excluded from analysis in round 1 and round 2.

We deployed the portal questionnaire in 3 steps to support iterative optimization (Figure 1). The portal notified patients that there was a new questionnaire in their portal through a general email or a pop-up on their smartphone app. Patients were required to have an active portal account and had to log into the account to complete the questionnaire. For both round 1 and round 2, we held our date and time fields constant by sending the message on Tuesdays at 10:20 AM Eastern Daylight Time (EDT).

##### SMS Text Message Survey

The text survey to assess smoking status consisted of 4 questions, which were either multiple-choice or open-ended (Table S1 and Figure S1 in Multimedia Appendix 1). This software would not allow for the restriction of numeric-only values. Conditional display of questions was not possible with the text survey platform, so all patients saw all questions. As a result, we added the leading text “If you stopped” to the question “How old were you when you stopped smoking?” We also evaluated response rates by message framing (Figure 1).

As in the portal questionnaire, we deployed the text survey using a 2-round phased approach to support iterative optimization (Figure 1). We randomly assigned patients to receive 1 of 3 messages: “gain,” “loss,” or “helpfulness” (Table 1). Patients were randomized in Excel (Microsoft Corporation) using the RAND function to assign each participant a random number and the RANK and ROUNDUP functions to evenly distribute participants in each of the 3 message groups. We sent an initial SMS text message on Tuesdays at 10:20 AM EDT, consistent with the portal questionnaire. Participants could either respond, not respond, or unsubscribe. We then sent a nudge or second message to all nonresponders who had not unsubscribed, such that 50% of the nonresponders received the same message as the index message, while 50% received a different message, split evenly among the other 2 framing options (Figure S3 in Multimedia Appendix 1 depicts the message trial schema). We sent the nudge message 2 days after the initial message on Thursday at 10:20 AM EDT.

### Statistical Approach

All statistical analyses were performed using R (version 4.1.0; The R Project for Statistical Computing). For analyses including 2 variables (portal vs text survey and same vs different second push messages), Fisher exact test was used. For comparison of framing, we performed a random effects logistic regression model for outcome of response and exposure of survey with random intercept for patients’ to account for repeated measures.

The primary outcome measure was the proportion of surveyed patients who responded based on survey modality (portal vs text survey). As a secondary outcome, we assessed response rates for the text survey based on framing and repeated pushes (first or second). Odds ratios (ORs) were calculated for each of these comparisons.

As an additional secondary outcome, we also compared the data obtained from survey responses to those already existing in the smoking history captured in EPIC. Using Fisher exact test, we compared the number of patients with complete smoking histories, defined as adequate information to determine LCS eligibility (pack-years and time since quitting). We also analyzed the concordance between EPIC data and the data gathered from completed surveys for LCS eligibility, smoking status, and pack-years reported. We used the Cohen κ coefficient to compare LCS eligibility and smoking status. We used a 2-way random effects intraclass correlation coefficient to compare agreement in reported pack-years between the EMR and completed surveys [25]. A level of significance of α=.05 was used.

## Results

### Survey Response Rates

Overall, the characteristics of responders and nonresponders for both survey modalities were similar, except that responders to the text survey were more likely to identify as White and to have stopped smoking compared with text nonresponders (Table 2).

**Table 2:** Characteristics of responders and nonresponders to the portal questionnaire and text survey.

			Portal questionnaire responders (n=35)		Portal questionnaire nonresponders (n=469)		Text survey responders (n=191)	Text survey nonresponders (n=809)
Age (years), mean (SD)			59.11 (8.6)		59.04 (6.9)		59.31 (6.66)	60.23 (7.77)
**Race or Ethnicity, n (%)**								
	Asian	1 (2.9)		7 (1.5)		2 (1.0)		8 (1)
	Black	17 (48.6)		259 (55.2)		99 (51.8)		530 (65.5)
	Hispanic or Latino	4 (11.4)		50 (10.7)		25 (13.1)		61 (7.5)
	White	12 (34.3)		127 (27.1)		56 (29.3)		177 (21.9)
	Other	1 (2.9)		6 (1.3)		1 (0.5)		11 (1.4)
	Declined	0 (0)		20 (4.3)		8 (4.2)		22 (2.7)
Male, n (%)			14 (40)		223 (47.5)		103 (53.9)	471 (58.2)
Individuals who reported current tobacco use (as recorded in EPIC), n (%)			13 (37.1)		189 (40.2)		69 (36.1)	397 (49.1)

**Figure 2:**
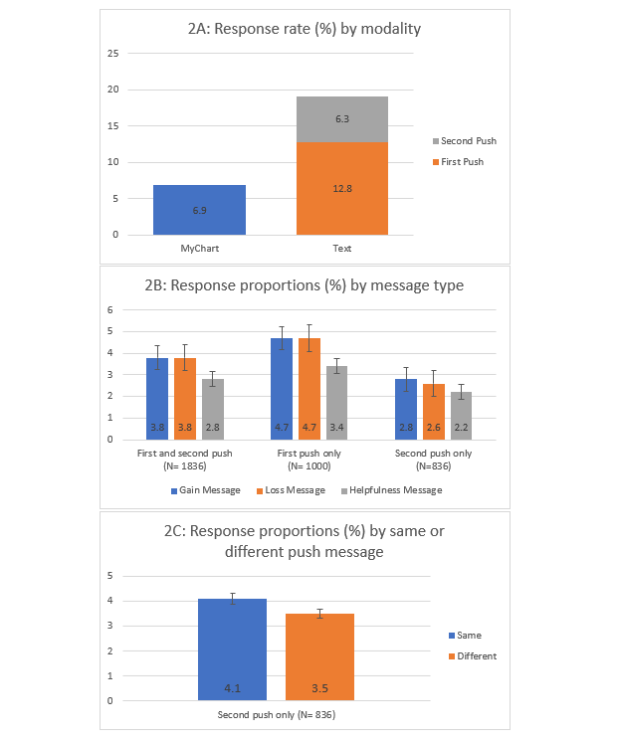
Response rate and proportions based on survey modality and message framing.

The response rate for the portal questionnaire was 6.9% (35/504) and the response rate for the SMS text message–based survey was 19.1% (191/1000) with an OR of 3.18 (95% CI 2.16-4.79; *P*<.05) (Figure 2). Across all survey rounds, patients were less responsive to the “helpfulness” message compared with the “gain” message (OR 0.29, 95% CI 0.08-0.99; *P*<.05) and compared with the “loss” message (OR 0.32, 95% CI 0.09-0.91; *P*<.05); however, there was no difference in responses between the “gain” and “loss” messages (OR 0.89, 95% CI 0.31-2.55, *P*=.82) (Figure 2). There was no significant difference in response rates to different message frames when comparing responses from only the first push survey round or responses from only the second push survey round. In reference to the first push message frame, there was also no difference in response rate if the same message framing or a different message framing was used in the second push (OR 1.3, 95% CI 0.74-2.17; *P*=.44). The overall unsubscribe rate for the text survey was 5.5% (55/1000). There was no significant difference in unsubscribe rates depending on the message framing.

### Completed Smoking History Data Analysis

Both the portal questionnaire and text survey were significantly more likely to obtain complete data compared to the available data in the medical chart (Table 3).

**Table 3:** Comparison of smoking history completeness to determine lung cancer screening (LCS) eligibility.

	EPIC data, n/N (%)	Survey data, n/N (%)	OR^a^ (CI)	*P* value
Portal questionnaire	93/504 (18%)	31/35 (88%)	34.2 (11.8-99.4)	<.05
Text survey	431/1000 (43.1%)	176/191 (89%)	6.8 (3.8-11.1)	<.05

^a^OR: odds ratio.

Of the responses that did not provide completed data, patients reported “do not remember” (n=0 in the portal and n=3 in the text), submitted nonnumeric answers such as “only tried briefly” (n=0 in the portal and n=7 in the text), or submitted answers that were extreme outliers (n=4 in the portal and n=5 in the text), leading to exclusion. Furthermore, 6 individuals who reported current smoking and responded to the text survey reported gaps in their smoking history. Since we could not discern whether these “gaps” represented periods where patients had stopped smoking versus incomplete data, we instead used current age to calculate total pack-years.

The portal questionnaire generated 24 newly complete smoking histories to determine LCS eligibility for those whose EPIC data were not complete. The number of patients who had complete data for both the existing information in EPIC and the new data obtained from the portal questionnaire was low (7 out of 504). This small sample size limited our statistical analysis of the concordance for LCS eligibility and current smoking status between the medical chart data and the data gathered in the portal questionnaire. However, our raw data demonstrates relative agreement, with 6 of 7 responses in agreement for LCS eligibility (eligible or ineligible) and 7 of 7 responses in agreement for smoking status (current or former). The average number of pack-years recorded in EPIC for this group was 10.82, and the average number of pack-years recorded by the portal questionnaire was 9.27, with a good correlation between the 2 sets of data (intraclass correlation [ICC] 0.81, 95% CI 0.28-0.96).

The text survey generated newly complete data to determine LCS eligibility for 89 patients, whose EPIC data were incomplete. Complete data to determine LCS eligibility in both the medical chart and the text survey were available for 87 patients. However, there was poor agreement between the existing data in the medical chart and those collected by the text survey. The 2 sets of data were discordant in identifying whether patients were eligible for LCS (Cohen κ 0.32, 95% CI 0.029-0.62) and in identifying current smoking status (Cohen κ 0.008, 95% CI –0.0077 to 0.024). The average number of pack-years for this group recorded in the medical chart was 14.83, and the average number of pack-years recorded by the text survey was 9.81, with a poor correlation between the 2 data sets (ICC 0.27, 95% CI 0.04-0.48).

## Discussion

Improving our health systems’ ability to capture accurate and complete smoking histories could have significant implications for the delivery of care, specifically for LCS and smoking cessation counseling. We found that a PGHD approach using patient-generated survey data is a feasible way to engage patients and collect smoking histories. Our trial provides a model for robust, pragmatic evaluation of digital interventions for quality improvement. We were able to test 2 types of survey delivery methods and 3 different survey message framings, all with significant equipoise in the literature. Overall, the portal questionnaire was less effective in generating responses compared to the text survey, and the “helpfulness” framing was less effective in generating responses compared to the “gain” and “loss” framings. A major finding was that both the text survey and the portal questionnaire generated more complete smoking histories to determine LCS eligibility when compared to the existing information available in the EMR.

Previous studies report a wide range of response rates to web-based surveys, with multiple factors contributing to decisions to respond, such as type of information collected, framing, number of reminders, and patients’ health care use [5,26-30]. Our SMS text message survey generated a response rate of 19.1% (191/1000), which is within the range reported in previous studies evaluating web-based surveys and significantly higher than that generated by the portal questionnaire [5,26-30]. The lower response rate to the portal questionnaire may have been influenced by the inability to test framing or deliver nudge messages. However, if we maintain the assumption of an equivalent increase in response rate due to framing (1.8%) and separately due to nudging (6.3%), then we can infer a response rate of 15% (compared to the actual response rate of 35/504, 6.9%). This demonstrates that while framing and a lack of nudge likely had a significant impact, other factors also contributed. One possible explanation is that while the text survey used an interruptive design of direct messaging that could be accessed immediately, the configuration of the portal messaging required access to an app or email and a separate login into the portal, making it less accessible.

Our findings demonstrated improved engagement with “gain” and “loss” framing as opposed to “helpfulness” framing. It is well documented that “gain” and “loss” framing improves patient engagement, attitudes, and motivation [31-36], which, based on our data, likely extends to patient engagement to report smoking history data. However, direct comparisons to “helpfulness” messaging are limited. In fact, the use of “helpfulness” messaging is better documented in nonmedical survey methodology [24]. One potential explanation for the superior performance of “gain” and “loss” framing compared with “helpfulness” framing is that the messages used for the “gain” and “loss” framing center on the implications of responding for the patient, while the “helpfulness” framing centers on the implications of responding for the health care provider. User-centered design has been shown to improve patient engagement [37-39]. Further evaluation of the reasons underlying differential engagement based on message framing, for example, with qualitative analysis, is needed in future studies.

As in this study, where the PGHD generated more complete smoking histories compared to the EMR, other studies have highlighted how dedicated structured data fields are missing key information needed to assess eligibility, such as packs per day, pack-years, and years since quitting [3-6,8,40]. In our health system, some individuals, such as medical assistants and tobacco treatment specialists, may update smoking history in structured fields, but many clinicians are more likely to document smoking history in unstructured notes. As a result, the structured data fields may be more vulnerable to becoming outdated over time and more likely to underreport smoking histories [4]. Survey instruments, in contrast, can be designed to force complete data entry and can be clarified with the patient in the context of a shared decision-making conversation. This could further alleviate completeness issues.

While the data generated from the surveys were more complete than the EMR, it is difficult to ascertain which history is most accurate. Previous research has found substantial agreement between self-reported smoking history comparing a baseline survey and a 1-month follow-up, with a higher likelihood of inconsistent reporting from individuals currently smoking as compared with individuals who have stopped smoking [41]. This reproducibility could be considered a proxy for accuracy. Other studies have treated the history obtained from shared decision-making as the source of truth. Modin et al [6] found a high degree of underreporting in the health record compared to the history obtained in a shared decision-making discussion. It is difficult to know whether the robustness of a nuanced history elicited by a trained clinician could produce a more true result or if other factors, such as time from the last history ascertainment, may influence accuracy. Which of the tobacco use histories is most accurate is particularly salient for the text survey, which demonstrated significant disagreement with the data in the EMR. While the portal questionnaire data suggested closer agreement with the EMR, this is likely an effect of the very small sample size of patients who had complete portal questionnaire data and EMR data. Future studies might use larger sample sizes and repeated measures to better ascertain the connection between completeness and accuracy.

The feasibility of web-based surveys to engage patients and obtain medical data has important implications for a LHS beyond tobacco use history. Consistent, easily accessible structured medical data can be used to target interventions in preventive and follow-up care, for example, the use of web-based symptom checkers to remotely triage patient concerns [42,43]. While innovations such as artificial intelligence and large language models are being proposed as a way to better use unstructured data, these technologies are not yet commercially available or integrated into the EMR and may be costly [44]. Using existing infrastructure, such as portal questionnaires and text surveys, is a low-cost, readily available way to asynchronously engage with patients and gather more structured data for clinical use.

This study was strengthened by testing multiple modalities and multiple message frames to determine the most effective method for engaging patients to self-report tobacco use history. However, this study has some limitations. The use of specific web-based technologies to obtain smoking history data and only English-language data may limit generalizability. Judgment sampling may introduce selection bias, which larger sample sizes would mitigate. However, the cohort characteristics would suggest a diverse cohort that was balanced across intervention groups. Furthermore, obtaining information on smoking history, regardless of modality, can be impacted by social desirability bias, recall bias, and recency bias, all of which may have contributed to the data obtained in this trial [4,45]. We were unable to test message frames in the MyChart Questionnaire portal system due to the unmodifiable global configuration of the initial email message. Also, the CareTour texting platform is designed for appointment reminder functionality. As we were using it for an alternate purpose, we were limited by the lack of conditional display and the inability to restrict data entry on field types, leading to the collection of unusable data. While this modality was able to capture dynamic histories of starting and stopping smoking, it was unclear how to easily translate this into a functionally detailed history. In fact, it remains unclear whether open-ended, “yes” or “no” questions would be most effective in capturing enough of a detailed smoking history to identify patients for interventions such as LCS [46]. Identifying the optimal questions and technology to navigate these limitations should be prioritized in future projects.

This study demonstrates that patients are likely to engage with a text-based survey using “gain” or “loss” framing to report detailed and complete smoking histories. Optimizing web-based surveys to collect tobacco use history and other medical data directly from patients is an appealing approach to improving health care delivery, especially if fully integrated into the EMR, as this could allow health care providers to proactively engage patients in LCS shared decision-making or smoking cessation counseling. Future work should focus on the validation of patient-generated history and the patient experience with receiving and completing a self-reported smoking history survey to allow for further optimization and implementation.

## Appendix

Multimedia Appendix 1 Images outlining the survey questions as they appeared to participants and trial schema.

## Abbreviations

EHR: electronic health record
EMR: electronic medical record
LCS: lung cancer screening
LHS: learning health system
PGHD: patient-generated health data
