# Supplementary material for: Efficacy of Digital Outreach Strategies for Collecting Smoking Data: Pragmatic Randomized Trial
Source: JMIR Form Res. 2024 Feb 9;8:e50465. doi: 10.2196/50465 (PMC10891497; doi:10.2196/50465)

Table S1: Comparison of Portal Questionnaire and Text Survey Questions

Portal Questionnaire	Text Survey
**Please select all that apply to describe your tobacco use history:**<br>● I stopped smoking cigarettes<br>● I currently smoke cigarettes<br>● I have never smoked cigarettes<br><br>**Individuals currently smoking only: How often do you smoke?**<br>● Every Day<br>● Some Days<br><br>**How old were you when you started smoking?**<br><br>**Individuals who formerly smoked only: How old were you when you stopped smoking?**<br><br>**How many cigarettes do/did you usually smoke in a day? (1 pack = 20 cigarettes)**<br><br>**What is the most cigarettes you have ever smoked in a day?**	**Please select the answer that describes your tobacco use history:**<br>● I stopped smoking cigarettes<br>● I currently smoke cigarettes<br>● I have never smoked cigarettes<br><br>**How old were you when you started smoking?** Please enter a number between 1-100 or enter "0" if you never smoked.<br><br>**How many cigarettes do/did you usually smoke in a day? (1 pack = 20 cigarettes)** Please enter a number between 1-100 or enter "0" if you never smoked.<br><br>**If you stopped, How old were you when you stopped smoking?** Please enter a number between 1-100 or enter "0" if you are a current smoker or never smoked.

**If you use something other than cigarettes, please select all that apply:**<br>● I smoke cigars/cigarillos<br>● I use e-cigarettes/vape every day<br>● I use e-cigarettes/vape some days<br>● I use chew<br>● I smoke tobacco pipes<br>● I use snuff - I have never used any tobacco products	

Figure S1: Text Survey Launched Via Web Link

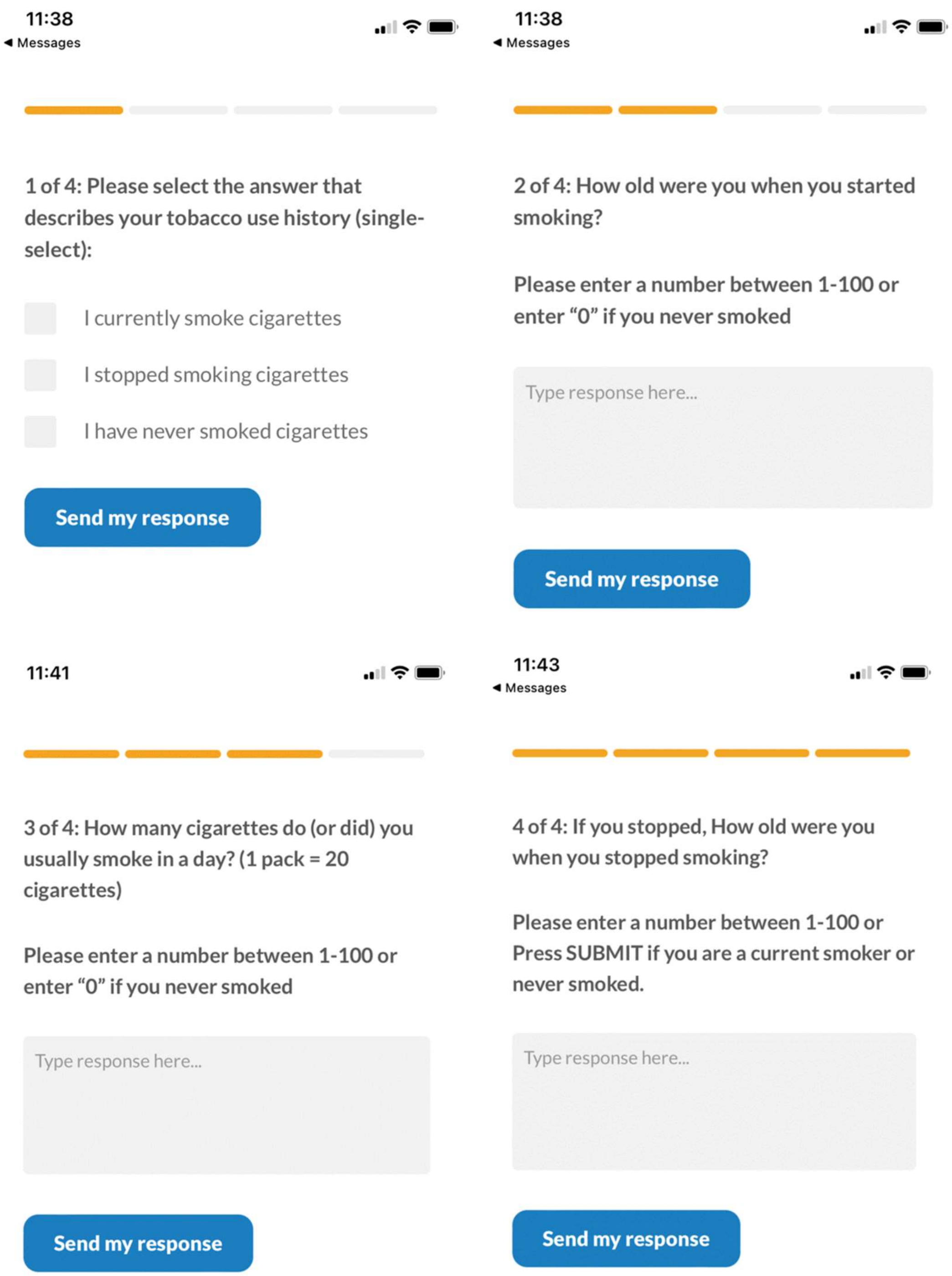

Figure S2: Portal Questionnaire

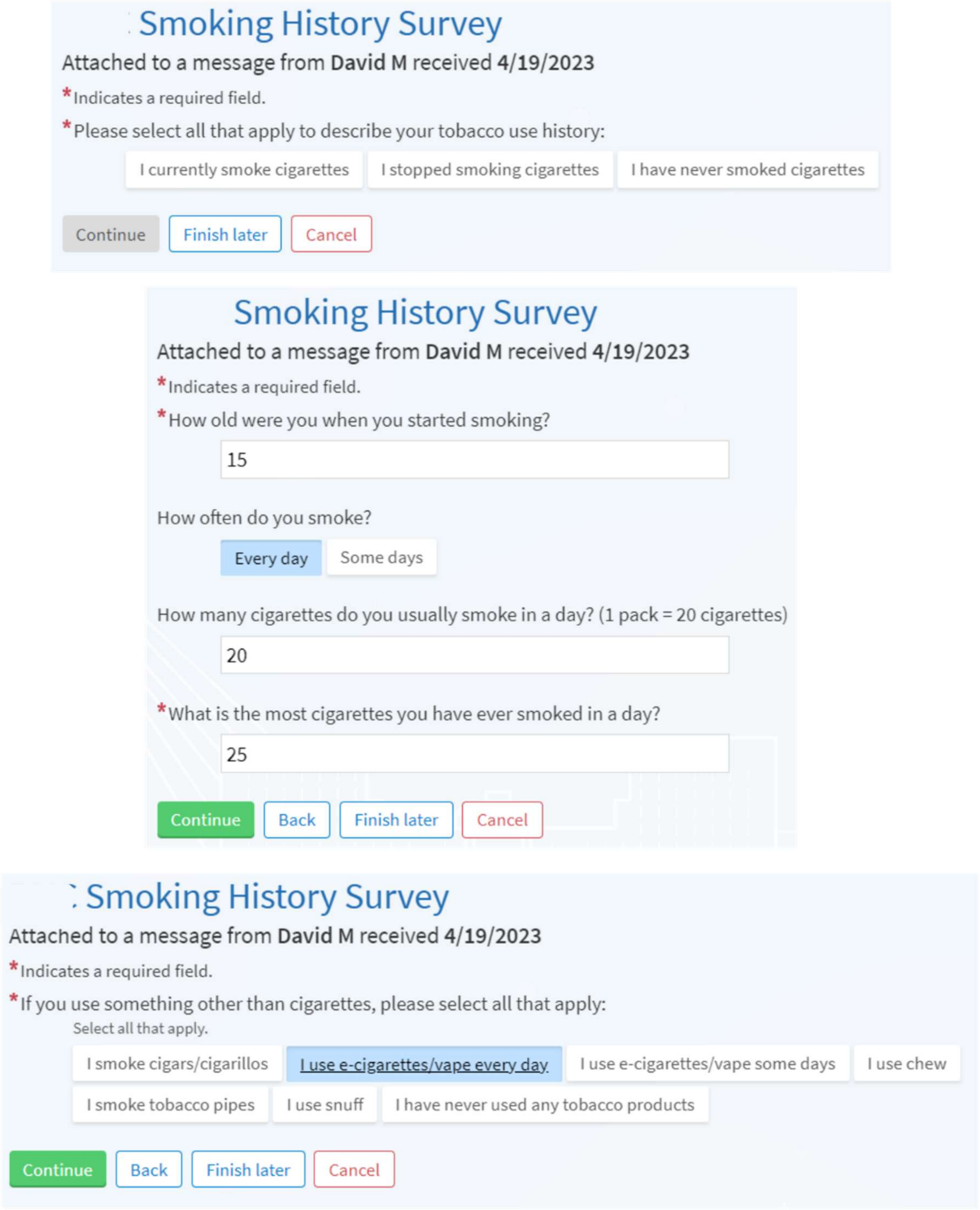

Figure S3: Message Trial Schema

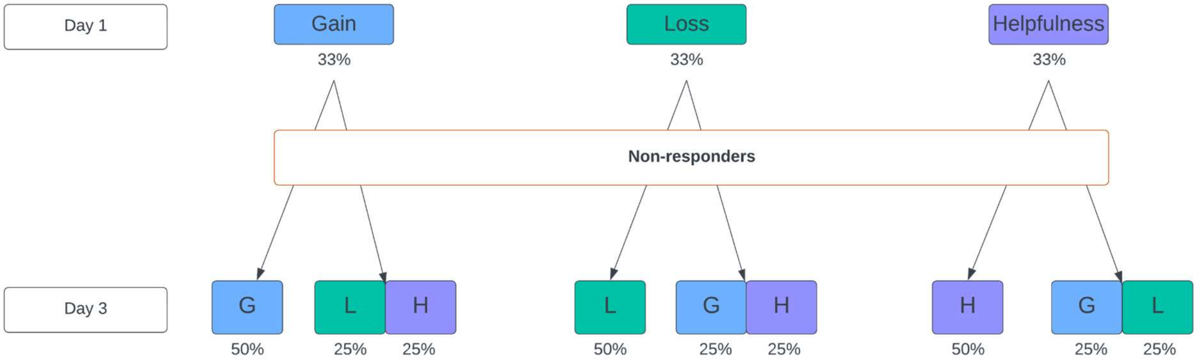

Supplement: Multimedia Appendix 1 [file formative_v8i1e50465_app1.pdf]
